# Fulminant Rheumatoid Vasculitis With Digital Necrosis and Cutaneous Ulcerations: A Case Report

**DOI:** 10.7759/cureus.103125

**Published:** 2026-02-06

**Authors:** Amner Sánchez Montenegro, Andrea Nicole Miranda Santamaría, Amanda Gabriela Cangas Isacaz, Alessandro Pichilingue Laos, Angel Francisco Cedillo Cedillo, Mariana Caballero Lagares

**Affiliations:** 1 Department of Disaster Management, Lambayeque Regional Hospital, Chiclayo, PER; 2 Department of Medicine, Evangelical University of El Salvador, San Salvador, SLV; 3 Department of Medicine, Central University of Ecuador, Quito, ECU; 4 Department of Medicine, José Faustino Sánchez Carrión National University, Huacho, PER; 5 Department of Medicine, University of Cuenca, Cuenca, ECU; 6 Department of Medicine, University of Sinú, Montería, COL

**Keywords:** digital necrosis, extra-articular manifestations, multiorgan failure, rheumatoid arthritis, rheumatoid vasculitis

## Abstract

Rheumatoid vasculitis (RV) is an uncommon but potentially fatal extra-articular manifestation of rheumatoid arthritis (RA), characterized by severe systemic inflammation and high mortality despite advances in disease-modifying therapies. We describe a 52-year-old woman with long-standing seropositive RA and irregular use of methotrexate who presented with progressive digital ischemia and extensive necrotic cutaneous ulcers, in the absence of active synovitis. Histopathology revealed small-vessel vasculitis, accompanied by markedly elevated rheumatoid factor and anti-cyclic citrullinated peptide titers, hypocomplementemia, and negative antineutrophil cytoplasmic antibodies. Despite high-dose corticosteroids, vasodilator therapy, and broad-spectrum antibiotics, the disease rapidly evolved to multiorgan involvement, including respiratory failure requiring mechanical ventilation, rapidly progressive renal failure requiring hemodialysis, and ischemic central nervous system lesions, culminating in refractory multiorgan failure and death. This case illustrates a fulminant presentation of RV with aggressive cutaneous and visceral involvement, emphasizing that severe vasculitis may occur independently of articular disease activity and is associated with poor prognosis. RV should be suspected in patients with long-standing RA who develop unexplained ischemic or ulcerative lesions, even in the absence of active arthritis. Early recognition and timely, sustained immunosuppression are critical to prevent irreversible organ damage and fatal outcomes.

## Introduction

Rheumatoid arthritis (RA) is a chronic inflammatory autoimmune disease that primarily affects peripheral joints in a symmetric pattern. Its global prevalence is estimated to range from 0.3% to 2.1%, with a higher incidence in women and a peak onset between ages 30 and 50 [1]. Although joint involvement is the hallmark of the disease, RA is a systemic condition that can produce significant extra-articular manifestations, which occur in a considerable proportion of patients and are associated with longer disease duration, high rheumatoid factor titers, and persistent inflammatory activity [2].

Rheumatoid vasculitis (RV) is one of the most severe extra-articular complications of RA and is associated with substantial morbidity and mortality, with reported five-year mortality rates reaching 40-50% [3]. RV is currently considered a rare condition, with an estimated annual incidence of approximately 12.5 cases per million inhabitants. Historically, it affected 1-5% of patients with RA; however, its incidence has markedly declined with the advent of early diagnosis and the widespread use of conventional and biologic disease-modifying antirheumatic drugs (DMARDs) [3,4]. Despite its reduced frequency, RV remains a life-threatening manifestation, and delayed recognition may result in irreversible tissue damage, severe infections, organ dysfunction, and significant functional impairment.

Herein, we report the case of a 52-year-old woman with long-standing seropositive RA and irregular methotrexate use who developed extensive digital necrosis and large ulcerative lesions of the lower extremities in the absence of significant articular activity. This case is particularly noteworthy because it illustrates that severe extra-articular manifestations, such as RV, may occur independently of active synovitis and lead to a potentially fulminant outcome.

## Case presentation

A 52-year-old woman presented to the emergency department with a one-month history of progressive digital cyanosis and necrosis of the left hand, accompanied by an ulcerated lesion with a necrotic center and foul odor in the right sural region. Her medical history was notable for RA diagnosed four years earlier, treated irregularly with methotrexate (15 mg/week with folic acid) and leflunomide (20 mg/day). At the time of presentation, she was taking methotrexate only, along with intermittent courses of systemic corticosteroids. She denied tobacco or alcohol use.

**Figure 1:**
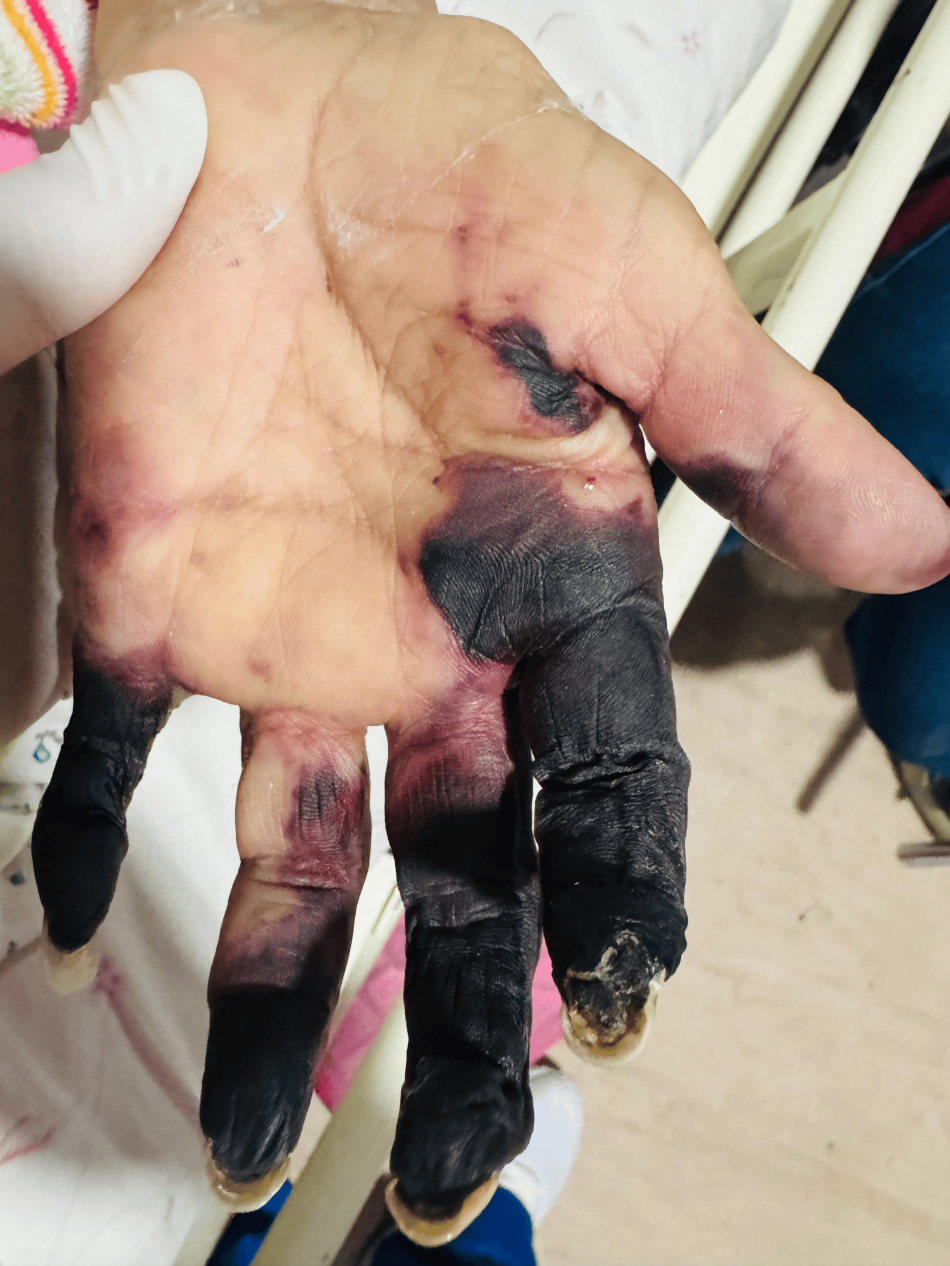
Palmar view of the left hand showing extensive digital ischemia and necrosis involving multiple fingers without documented clinical evidence of neuritis

**Figure 2:**
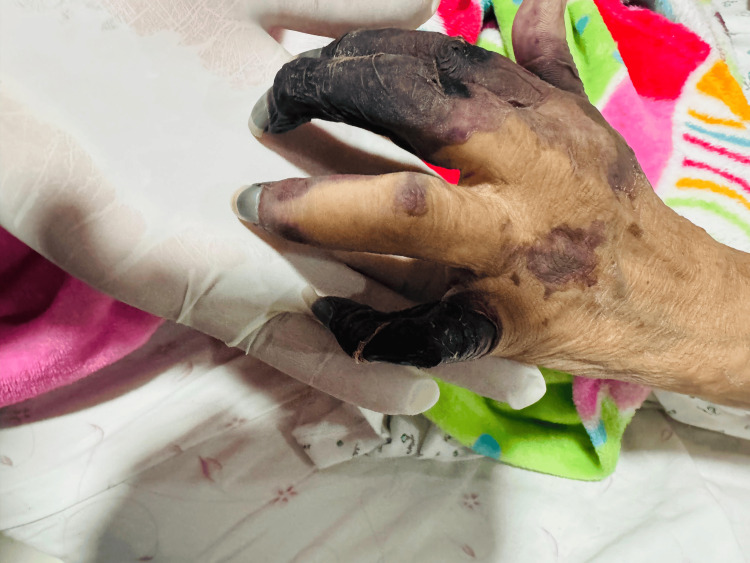
Dorsal view of the same hand demonstrating widespread necrosis and ischemic skin changes consistent with advanced vascular compromise

**Figure 3:**
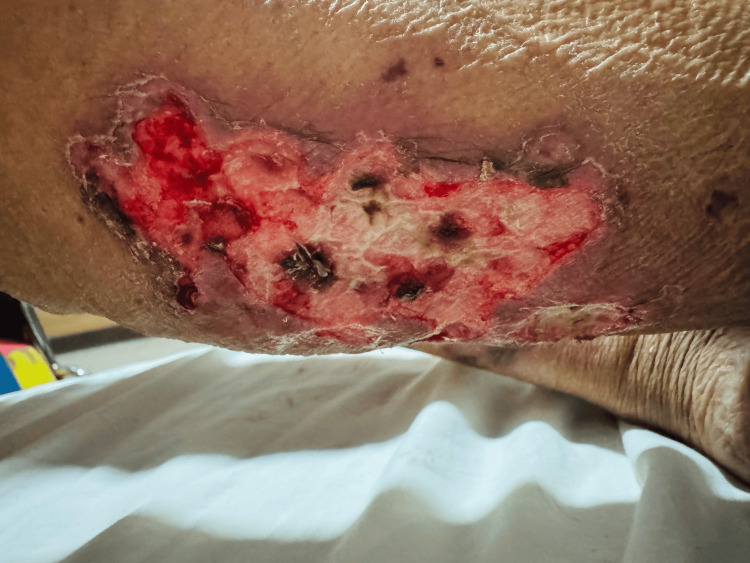
Deep necrotic ulcer in the right sural region after surgical cleansing and debridement illustrating severe ischemic cutaneous involvement

On admission, her vital signs were stable. Physical examination revealed dry necrosis of the second through fifth fingers of the left hand (Figures 1-2). Additional findings included a 2-cm crusted lesion with a hemorrhagic scab on the left elbow, a 6-cm necrotic ulcer in the right sural region (Figure 3), and a 4-cm necrotic ulcer in the right lateral supramalleolar region. Two smaller necrotic ulcers were also identified on the toe pads of both feet. No joint deformities, active synovitis, or involvement of the right hand were observed. No history or clinical evidence of Raynaud's phenomenon was documented.

On admission, the patient was hospitalized under the internal medicine service, and an urgent rheumatology consultation was requested. An extended immunologic profile was obtained. Additional evaluations by gynecology, dentistry, and otolaryngology revealed no evidence of involvement of other organ systems. The orthopedics service recommended conservative management of the left-hand lesions and daily local care of the right sural ulcer.

**Figure 4:**
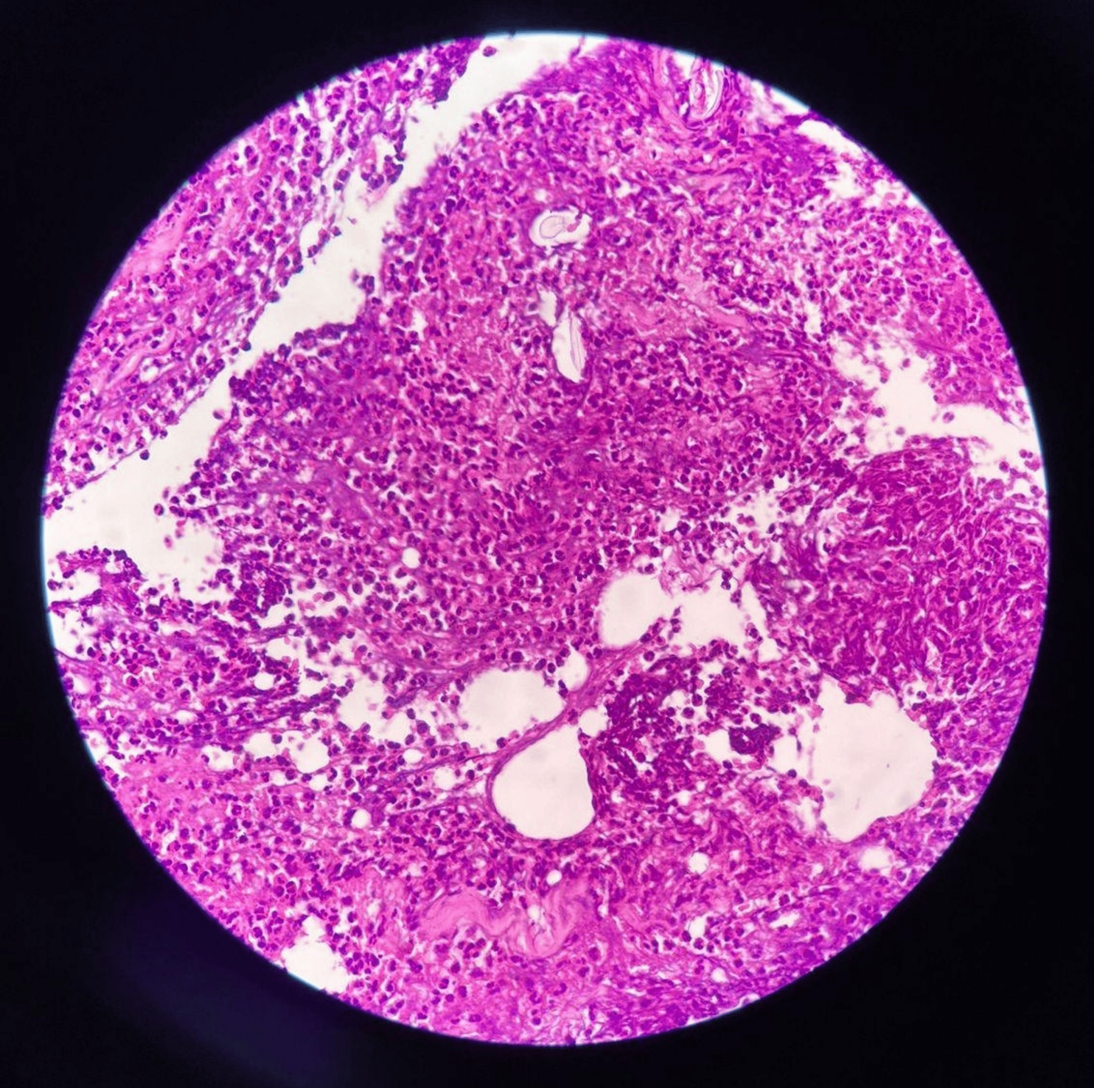
Skin biopsy showing necrotizing small-vessel vasculitis with dense perivascular and transmural inflammatory infiltrate and focal fibrinoid necrosis (hematoxylin and eosin stain ×200)

Histopathological examination of a skin biopsy demonstrated a neutrophilic inflammatory infiltrate involving small vessels, moderate infiltration of the adipose tissue, and marked inflammatory involvement of connective tissue with necrosis, findings consistent with small-vessel vasculitis (Figure 4). Arterial and venous Doppler ultrasound of the upper and lower extremities showed preserved blood flow with no evidence of thrombosis. Transthoracic echocardiography revealed left ventricular dilation with a reduced ejection fraction of 40%.

Laboratory evaluation showed markedly elevated inflammatory markers, hypocomplementemia with low C3 and C4 levels, and significantly increased titers of rheumatoid factor and anti-cyclic citrullinated peptide antibodies, with a negative antineutrophil cytoplasmic antibody (ANCA) test (Table 1). Based on the clinical presentation, histopathological findings, and immunologic profile, a diagnosis of systemic RV was established. Given the presence of digital ischemia and necrotic ulcers, alternative etiologies were considered. Antiphospholipid antibody testing was negative (Table 1), ANCA testing was negative, Doppler ultrasound demonstrated preserved arterial flow without thrombosis, and the clinical context of long-standing seropositive RA with hypocomplementemia supported immune-complex-mediated rheumatoid vasculitis over thrombotic or ANCA-associated vasculitic processes.

**Table 1:** Laboratory tests and findings at admission and during hospitalization p-ANCA: perinuclear antineutrophil cytoplasmic antibodies, c-ANCA: cytoplasmic antineutrophil cytoplasmic antibodies

Parameter	Value	Unit	Reference range
White blood cell count	13,960	cells/µL	4,500-10,000 cells/µL
Hemoglobin	10.3	g/dL	12-16 g/dL
Platelet count	572,000	platelets/µL	150,000-450,000 platelets/µL
Blood urea nitrogen (BUN)	141.24	mg/dL	7-20 mg/dL
Creatinine	5.04	mg/dL	0.6-1.3 mg/dL
Anti-cyclic citrullinated peptide antibody (anti-CCP)	909.4	U/mL	<5.0 U/mL
C-reactive protein (CRP)	127.26	mg/L	<3 mg/L
Erythrocyte sedimentation rate (ESR)	57	mm/h	<30 mm/h
Rheumatoid factor (RF)	>900	IU/mL	<15 IU/mL
Complement C3	74	mg/dL	90-180 mg/dL
Complement C4	15	mg/dL	10-40 mg/dL
Antinuclear antibody (ANA)	Negative	Titer	Negative: <1:40, weakly positive: 1:40-1:80, positive: ≥1:160
Anti-Smith antibody	1.2	U/mL	Negative: <15.0 U/mL, positive: >25.0 U/mL
p-ANCA	Negative		Negative
c-ANCA	Negative		Negative
Anti–β2-glycoprotein IgM	1.7	U/mL	Negative: <5.0, indeterminate: 5.0-8.0, positive: >8.0
Anti–β2-glycoprotein IgG	2.5	U/mL	Negative: <5.0, indeterminate: 5.0-8.0, positive: >8.0
Anti-cardiolipin IgA	1.8	U/mL	Negative: <10.0, positive: >10.0
Anti-cardiolipin IgG	5.8	U/mL	Negative: <10.0, positive: ≥10.0
Anti-cardiolipin IgM	8.4	U/mL	Negative: <10.0, positive: ≥10.0
Anti–β2-glycoprotein (total)	1.1	U/mL	Negative: <5.0, indeterminate: 5.0-8.0, positive: >8.0
Anti-phosphatidylserine IgG	10.2	GPL-U/mL	Negative: <10.0, positive: >10.0
Anti-phosphatidylserine IgM	15.4	MPL-U/mL	Negative: <10.0, positive: >10.0
Anti-prothrombin IgG	3.7	U/mL	Negative: <10.0, positive: >10.0
Anti-prothrombin IgM	2.8	U/mL	Negative: <10.0, positive: ≥10.0
24-hour proteinuria	587.43	mg/day	<150 mg/day

Treatment was initiated with high-dose intravenous methylprednisolone pulses (1 g/day for three consecutive days), in combination with vasodilator therapy including sildenafil (50 mg/day) and nifedipine (30 mg every 12 hours). Given the presence of necrotic cutaneous lesions and concern for secondary infection, empirical broad-spectrum antibiotic therapy was initiated with piperacillin-tazobactam for four days, followed by escalation to meropenem (1 g every eight hours) and vancomycin (1 g every 12 hours).

**Figure 5:**
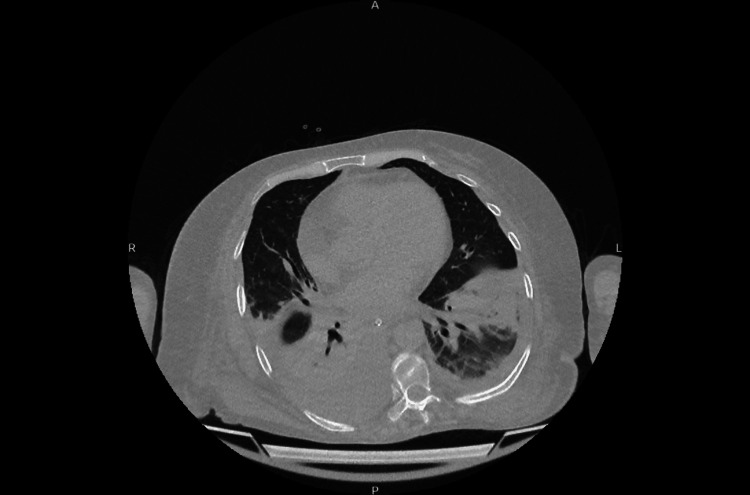
An axial computed tomography scan of the chest without contrast showing bilateral pleural effusions predominantly on the right and compressive atelectasis of the lower lobes, associated alveolointerstitial opacities, and inflammatory thickening of the chest wall extending from the pleural space

During the first week of hospitalization, the patient developed progressive cough and dyspnea. A pulmonology evaluation was obtained, and a non-contrast chest computed tomography scan revealed an alveolo-interstitial pneumopathy with bilateral pleural effusions, as well as inflammatory involvement of the thoracoabdominal wall suggestive of empyema necessitatis (Figure 5). Supplemental oxygen therapy, bronchodilators, and continuation of broad-spectrum antibiotic therapy were indicated.

**Figure 6:**
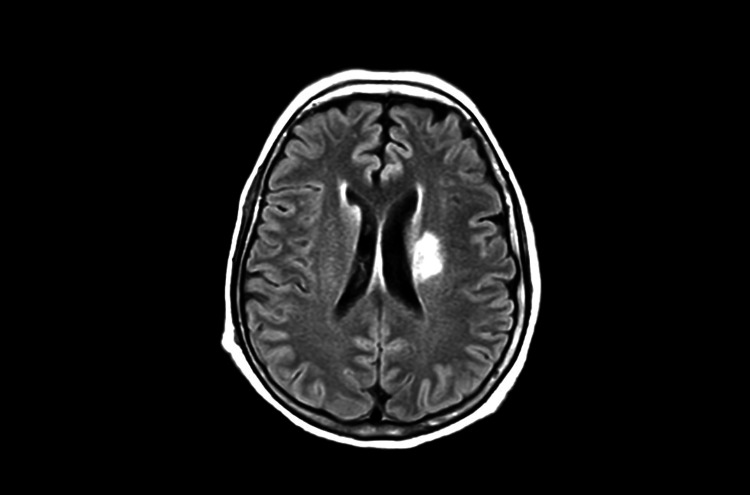
Axial FLAIR brain MRI showing focal hyperintense lesions in the left periventricular white matter compatible with ischemic changes involving deep cerebral structures FLAIR: fluid-attenuated inversion recovery, MRI: magnetic resonance imaging

Approximately two weeks after admission, the patient developed rapidly progressive acute kidney injury, classified as acute kidney injury network stage II, consistent with autoimmune glomerulopathy (urea: 141.24 mg/dL; creatinine: 5.04 mg/dL), which required initiation of intermittent hemodialysis. Subsequently, she developed acute confusion, prompting neurological evaluation. Brain magnetic resonance imaging demonstrated ischemic lesions involving the basal ganglia, consistent with central nervous system involvement (Figure 6). Given the evidence of progressive multiorgan involvement, rheumatology reassessed the patient and considered initiation of cyclophosphamide therapy. However, escalation of immunosuppression was deferred due to active infection, underscoring the therapeutic challenge of balancing disease control and infectious risk in severe systemic RV.

Subsequently, the patient was transferred to the intensive care unit due to progressive multiorgan failure and worsening respiratory compromise requiring invasive mechanical ventilation. Despite maximal supportive care, her clinical condition continued to deteriorate.

A new evaluation by the trauma surgery service determined that amputation of the left hand was indicated because of extensive necrosis; however, the procedure was deferred after discussion with the patient’s family. As part of intensive management, broad-spectrum antibiotic therapy was administered for 21 days with meropenem and for 14 days with vancomycin, adjusted for renal replacement therapy, along with systemic corticosteroid therapy with methylprednisolone at 100 mg every eight hours.

Despite these interventions, the patient showed no clinical improvement and progressed to refractory multiorgan failure. She ultimately died after 12 days of intensive care hospitalization.

## Discussion

This case describes a 52-year-old patient with a history of RA who developed RV, one of the most severe extra-articular complications of this disease [5]. In the current era of DMARDs, particularly biologic agents, the incidence of RV has decreased substantially, making severe systemic presentations uncommon but clinically significant due to their high morbidity and mortality [6].

The patient’s serological profile is fully consistent with the established risk factors for this condition, especially long-standing seropositive RA, which is strongly associated with the development of necrotizing vasculitis. Markedly elevated titers of rheumatoid factor (>900 IU/mL) and anti-cyclic citrullinated peptide antibodies (909 U/mL), together with the presence of hypocomplementemia, support a pathophysiological mechanism mediated by excessive formation of circulating immune complexes, with complement activation and systemic vascular deposition [7]. This process reflects the transition from a predominantly articular disease to an uncontrolled systemic inflammatory manifestation characteristic of severe RV. Histologic findings in RV range from involvement of small-sized vessels to medium-sized necrotizing arteritis, resulting in progressive tissue ischemia and multisystem organ damage [8].

Clinically, this case is distinguished by the severity of the cutaneous manifestations. Although RV most commonly presents with palpable purpura and superficial ulcers, this patient developed extensive digital necrosis involving multiple fingers, as well as large, deep necrotic ulcers in the sural region. This pattern of involvement is considered a severe form of the disease and is indicative of rapidly progressive vasculitis with a high risk of poor outcomes. Extensive digital necrosis, in particular, has been described in the literature as a marker of aggressive systemic vasculitis and unfavorable prognosis [9].

The most concerning finding was the involvement of vital organs. The patient developed rapidly progressive acute kidney injury requiring hemodialysis, raising strong concern for immune complex-mediated glomerulonephritis. Although a renal biopsy was not performed, the combination of rapidly progressive renal failure, complement consumption, and evidence of systemic vasculitis strongly suggested immune complex-mediated glomerular vasculitis. Such visceral involvement has consistently been associated with adverse outcomes in patients with RV [10].

A particularly relevant clinical finding was the absence of active arthritis at hospital admission, despite intense systemic disease activity. This dissociation between articular inflammation and extra-articular activity has been described in severe forms of RV. It reinforces the concept that vasculitis may represent an autonomous systemic manifestation of the underlying disease. In this setting, ischemic cutaneous lesions may constitute an early warning sign of widespread vascular involvement, even in the absence of overt synovial activity [11].

The treatment of severe RV is usually based on high-dose corticosteroids, combined with conventional immunosuppressive agents such as cyclophosphamide or biologic agents such as rituximab, which have been shown to improve disease activity and allow steroid dose reduction in a considerable number of cases [12]. In this case, corticosteroid pulses were initiated; however, the clinical course underscores the need for timely, sustained immunosuppression to prevent progression of tissue damage and relapse and highlights the therapeutic complexity posed by concomitant infections or conditions that may preclude early use of definitive immunosuppressive therapy.

Overall, this case illustrates a fulminant and atypical presentation of RV in a patient with long-standing seropositive RA and irregular disease control. Massive immune complex formation and complement activation likely contributed to systemic vascular injury, explaining both the ischemic cutaneous manifestations and the severe visceral involvement, including acute renal failure and ischemic injury of the central nervous system. Despite its current rarity, RV remains a highly lethal extra-articular manifestation of RA, underscoring the importance of early clinical suspicion, strict treatment adherence, and prompt multidisciplinary management in patients with long-standing RA who present with unexplained ischemic or ulcerative lesions.

## Conclusions

RV is a rare but highly lethal extra-articular complication of RA that may present with severe ischemic skin lesions and rapid multiorgan involvement, even in the absence of active synovitis. This case highlights that markedly elevated rheumatoid factor and anti-CCP titers with hypocomplementemia should prompt urgent evaluation for immune-complex-mediated vasculitis when digital ischemia or necrotic ulcers occur. Early recognition, strict adherence to disease-modifying therapy, and timely initiation of sustained immunosuppression are critical to prevent irreversible tissue damage. However, management is frequently complicated by concomitant infection, underscoring the need for close multidisciplinary coordination to balance immunosuppressive control of vasculitis against infectious risk.
